# Relevance between Proximal Humeral Migration and Rotator Cuff Tears

**DOI:** 10.3389/fsurg.2022.903538

**Published:** 2022-05-05

**Authors:** Yichong Zhang, Jianhai Chen, Mingtai Ma, Jiabao Ju, Baoguo Jiang, Peixun Zhang

**Affiliations:** Department of Trauma and Orthopedics, Peking University People’s Hospital, Beijing, China

**Keywords:** rotator cuff tear, proximal humeral migration, upward migration index, predictor, acromion humeral distance

## Abstract

**Background:**

Proximal humeral migration is common in patients with rotator cuff tears. In this study, we aimed to evaluate the relevance between proximal humeral migration and some rotator cuff tear-related factors.

**Methods:**

A total of 75 patients with unilateral rotator cuff tears were retrospectively included from August 2016 to January 2018 who underwent magnetic resonance imaging and X-ray examinations before enrollment. We introduced the upward migration index (UMI) to stratify the patients into three groups, Group A: 1 < UMI ≤ 1.3; Group B: 1.3 < UMI ≤ 1.4; and Group C: UMI > 1.4. Pearson correlation analysis and logistic regression analysis were used to evaluate the relationship between UMI and age, sex, body mass index, pain, fatty degeneration grade, tear size, and thickness of ruptured tendon. Then, the *χ*^2^ test and receiver operator characteristic curve were applied to measure the diagnostic value of UMI.

**Results:**

The average UMI was 1.34 ± 0.07, ranging from 1.16 to 1.48. For the Pearson correlation analysis, there was a negative correlation between UMI and tear size (*R* = −0.68, *p* < 0.01), and also, there was a negative correlation between UMI and the visual analogue scale score (*R* = 0.342, *p* < 0.01). What is more, there was a negative correlation between UMI and the fatty degeneration grade (*R* = −0.373, *p* < 0.01). Ordinal multinomial logistic regression analysis indicated that tear size (*β* = −1.825, *p* < 0.001) was the independent predictor of UMI, which was a risk factor for humeral upward migration. The cutoff points of UMI were 1.38 and 1.3 to determine tears and distinguish large tears from small ones.

**Conclusions:**

UMI is a good predictor for humeral upward migration, which is related to the tear size of posterosuperior cuff tears. When the UMI is <1.3, a large tear should be alerted. Combining physical examination and X-ray is helpful for evaluating rotator cuff tears.

## Introduction

Proximal humeral migration is common in rotator cuff tear patients, but the detailed mechanism is still unknown (1, 2). The possible reason includes deltoid pulling or spacing missing after rotator cuff tears. Currently, proximal humeral migration mainly depends on the direct measurement of the acromion humeral distance (AHD), which helps shoulder surgeons to evaluate tear severity and make treatment decisions (3). Previous studies have shown that AHD < 7 mm indicated a rotator cuff tear (4, 5). However, the traditional AHD is thought inaccurate in judging humeral migration (6) because the size of the humeral head varies among different genders, ages, heights, weights, and races. So, we introduced the upward migration index (UMI), which was first mentioned in 1996 (7), to evaluate the proximal humeral migration and eliminate the impact of anatomical differences and the magnifying effect of X-rays on subacromial space. Also, we proposed to find out the relationship between the degree of proximal humeral upward migration and other factors, such as tear size and fatty degeneration grade.

## Materials and Methods

### General Information

This study included patients with unilateral rotator cuff tears from Peking University People’s Hospital from August 2016 to January 2018. All the patients had shoulder pain before treatment. The posterosuperior cuff tears (supraspinatus and infraspinatus) were highly suspected by the physical examination and confirmed by further imaging examination. The inclusion criteria included the following: (1) Magnetic resonance imaging (MRI) of the affected shoulder was performed within half a year before inclusion and confirmed a full-thickness tear of the supraspinatus/infraspinatus with or without tendon retraction. (2) An X-ray examination was performed within half a year before inclusion. (3) The interval between the two examinations should not be longer than 1 month. Additionally, the exclusion criteria were the following: (1) a history of shoulder surgery on the same side; (2) a history of proximal humeral or acromial fractures on the same side; (3) a history of instability or dislocation; (4) tear of subscapularis muscles; and (5) a history of nerve injury on the same limb. Eventually, 75 patients met the criteria, including 38 males and 37 females, with an average age of 64.5 years (range: 35–87 years). The body mass index (BMI) was also recorded. All the patients signed informed consent before enrollment. Fifty-two patients without rotator cuff tears were also included for further comparison.

A standard protocol anteroposterial radiograph was taken of all patients, slightly turned to the image side (20°), and the arm in external rotation with the palm facing forward. The film-focus distance was measured at 115 cm, and a 15° craniocaudal tilt was used to project the undersurface of the acromion perpendicular (8, 9).

**Figure 1:**
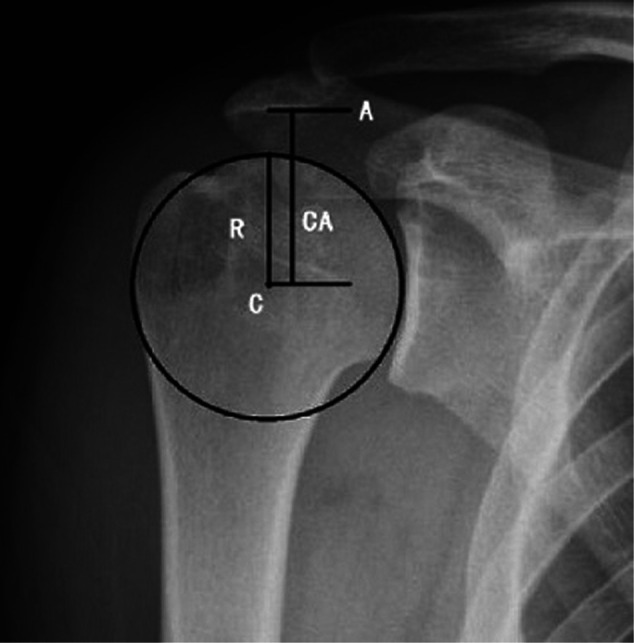
Radius of the humeral head (*R*) and CA distance were measured on an X-ray. UMI = CA/*R*.

All the data were extracted from the electronic database of Peking University People’s Hospital. Proximal migration was measured using the UMI, where the distance between the center of the humeral head and the undersurface of the acromion (CA) was divided by the radius of the humeral head (*R*) (**Figure 1**) (9). All the data were measured by orthopedic surgeons trained in shoulder surgery. Finally, patients were assigned into three groups according to the UMI value, namely, Group A: 1 < UMI ≤ 1.3; Group B: 1.3 < UMI ≤ 1.4; and Group C: UMI > 1.4.

### Evaluation of the Shoulder Pain

The visual analogue scale (VAS) was applied for pain assessment. Clinically, the total score is 10, with 0 for no pain, 1–3 for mild pain, 4–6 for moderate pain, 7–9 for severe pain, and 10 for unbearable pain.

### Magnetic Resonance Imaging

All patients underwent MRI of the shoulder in oblique coronal, oblique sagittal, and axial planes on a 3.0-T Signa scanner (GE Healthcare, Chicago, USA). The size, location, and degeneration degree of the supraspinatus/infraspinatus were estimated through the different planes of MRI. The tear size was obtained on the T2 oblique coronal and oblique sagittal planes with the maximal defect (10, 11). To measure the thickness of teared tendon thickness, the maximal stump of supraspinatus/infraspinatus on the T2 oblique coronal plane. The extent of muscle degeneration was defined as described by Goutallier et al. (12). Grade 0 indicated normal muscle, grade 1 was some fatty streaks, grade 2 was defined as <50% fatty muscle atrophy, grade 3 was 50% fatty muscle atrophy, and grade 4 indicated >50% fatty muscle atrophy. The Goutallier classification was originally intended to describe the performance of computed tomography and expanded later by Fuchs et al. to describe MRI results (13).

### Data Analysis

The acronyms are described in **Table 1**. SPSS Statistics (version 22.0, IBM) was used to analyze data in this study, and the Pearson correlation was used to determine the relationship between the MRI results, other different factors, and UMI grades.

**Table 1:** Baseline statistics of patients.

Variable (*n *= 75)	Variable name	Mean	Standard deviation	Minimum	Maximum
Age	Age	64.48	11.71	35.00	87.00
Body mass index	BMI	24.33	2.83	18.70	30.10
Humeral center to acromion	CA	30.95	2.75	23.34	37.53
Radius of humeral head	R	23.12	2.05	18.54	28.10
UMI value	UMI	1.34	0.07	1.16	1.48
UMI class	UMI_Class	2	0.77	1.00	3.00
Pain score	VAS	5.24	2.36	1.00	10.00
Supraspinatus fatty grade	*X*_1_	1.72	1.2	0	4.00
Tendon thickness at the tear site	*X*_2_	9.22	2.44	4.60	15.60
Tear area of supraspinatus	*X*_3_	297.47	311.86	21.24	1266.83

Considering that UMI may be affected by multiple factors, the factors associated with the UMI grade were selected as the independent variables in this study. The UMI grade was regarded as the dependent variable for further ordinal multinomial logistic regression to determine the possible influence and significance.

The receiver operator characteristic (ROC) curves were drawn, with the UMI values defined as the test variables and tear sizes defined as the state variables, to determine the cutoff point of tear sizes. Finally, ROC curves were drawn by combining the UMI values of 52 patients without tears together with the values of patients suffering tears as the test variables and the tear state as the state variables to obtain the cutoff value of the normal UMI grade and so on, which is of great significance for future diagnosis and treatment.

## Results

### Calculation of Upward Migration Index

According to the method described above, the CA and *R* values were obtained by the X-ray imaging system. The range of UMI values was 1.16–1.48, with a mean value of 1.34. According to the method defined previously, the patients were divided into three groups. There were 22 patients in group A, 31 patients in group B, and 22 patients in group C.

### Scores of Shoulder Pain on the Affected Side of Each Group

The VAS score was obtained from all the 75 patients and ranged from 1 to 10, with an average of 5.24. The mean score of group A was 5.9 (range: 1–10), group B was 5.8 (range: 2–10), and group C was 3.8 (range: 1–3).

### Comparison of Magnetic Resonance Imaging Results of Supraspinatus/Infraspinatus Tear between Different Groups

The size of tear is measured in the oblique sagittal and oblique coronal planes with the largest defect. The mean tear size was 297.47 mm^2^ (range: 21.24–1266.83 mm^2^). In group A, the mean tear size was 631.58 mm^2^ (range: 68–1266.83 mm^2^). For group B, the mean tear size was 196.23 mm^2^ (range: 31.68–801 mm^2^). In group C, the mean tear size was 105.98 mm^2^ (range: 21.24–348.80 mm^2^). Because of measurements in millimeters, the variance between different values seemed significantly magnified. So, all the statistical data were converted to the natural logarithmic (ln) and marked as Z3. The tendency of fatty degeneration in supraspinatus/infraspinatus (Goutallier grade 2 or above) was detected in 39 of 75 patients, which concludes 19 cases of grade 2, 13 cases of grade 3, and 7 cases of grade 4. Others included 12 cases of grade 0 and 24 cases of grade 1. The results are listed in **Table 2**. The mean thickness of the tendon was 9.1 mm (range: 4.6–11.7 mm) in group A, 9.5 mm (range: 5.7–14.7 mm) in group B, and 10.1 mm in group C (range: 6.3–15.6 mm).

**Table 2:** Comparison of the fatty degeneration of rotator cuff among different groups.

UMI classification	Goutallier classification				
	0	1	2	3	4
Group A (*n* = 22)	1	3	4	5	3
Group B (*n* = 31)	4	12	9	3	2
Group C (*n* = 22)	5	8	4	3	1

### Correlation between General Data and the Upward Migration Index of the Humeral Head

Pearson correlation analysis was performed between UMI and other factors in this study, and the results are listed in **Table 3**. The UMI grade was negatively correlated with the pain score (*R* = −0.342, *p* = 0.003). In contrast, the UMI grade had no correlation with age, gender, and BMI, which further proved that the index could minimize the influence of age, gender, height, and some other factors.

**Table 3:** Pearson analysis between different factors (the number at the top of each unit is a correlation coefficient, and the value below is a *p*-value). Gender: male = 1, female = 2.

3 Factors 6 UMI	Sex	Age	BMI	VAS	UMI class	CA	*R*
Sex	1.00000	0.22981^*^	−0.607^*^	0.084	0.13927	−0.40629^*^	−0.46452^*^
		0.0473	<0.0001	0.489	0.2334	0.0003	<0.0001
Age	0.22981^*^	1.00000	−0.173	0.055	−0.13019	−0.22163	−0.10017
	0.0473		0.138	0.639	0.2656	0.0560	0.3925
BMI	−0.607^*^	−0.173	1.00000	−0.157	0.129	0.295^*^	0.192
	<0.0001	0.138		0.178	0.271	0.01	0.098
VAS	0.084	0.055	−0.157	1.00000	−0.342^*^	−0.286^*^	−0.066
	0.489	0.639	0.178		0.003	0.013	0.571
UMI class	0.13927	−0.13019	0.129	−0.342^*^	1.00000	0.23950^*^	−0.33356^*^
	0.2334	0.2656	0.271	0.003		0.0385	0.0035
CA	−0.40629^*^	−0.22163	0.295^*^	−0.286^*^	0.23950^*^	1.00000	0.80288^*^
	0.0003	0.0560	0.01	0.013	0.0385		<0.0001
*R*	−0.46452^*^	−0.10017	0.192	−0.066	−0.33356^*^	0.80288^*^	1.00000
	<0.0001	0.3925	0.098	0.571	0.0035	<0.0001	

*Significance level was below 0.05.

### Correlation between Different Magnetic Resonance Imaging Results and Upward Migration Index

The correlation between different MRI results and UMI was presented separately (**Table 4**). Pearson correlation analysis confirmed a highly negative correlation between the logarithm of the UMI grade and rotator cuff tear area (*R* = −0.68, *p* < 0.001). For the UMI grade and fatty degeneration degree of the rotator cuff, the correlation analysis also showed a negative correlation (*R* = −0.373, *p* = 0.001). However, there was no correlation between the UMI grade and tendon thickness at the tear side (*R* = 0.15, *p* = 0.2017).

**Table 4:** Pearson correlation analysis between different MRI results and UMI.

UMI	Tear size logarithm (*Z*_3_)	Fatty degeneration grade (*X*_1_)	Tendon thickness (*X*_2_)
Correlation coefficient	−0.6814	−0.373	0.14911
Significance (bilateral)	<0.001	0.001	0.2017

### Ordinal Multinomial Logistic Regression Analysis

According to the previous analysis, the pain score, tear area, and fatty degeneration were correlated with the UMI grade. Further, ordinal multinomial logistic regression analysis was performed by taking the VAS score, fatty infiltration degree, and tear area of supraspinatus/infraspinatus (logarithmic value) as independent variables and the UMI grade as the dependent variable (**Table 5**). The goodness-of-fit test verified the validity of the model with *χ*^2^ = 65.6 (*p* < 0.001). **Table 5** shows that the odds ratio value was 6.2 (*p* < 0.001), so the larger the tear of the rotator cuff, the lower the grade of UMI, and the more severely the proximal humerus migrates.

**Table 5:** Ordinal multinomial logistic regression analysis of factors affecting proximal humeral migration.

Influence factor	OR	SE	Wald	DOF	*p*-value	95% CI	
						Lower limit	Upper limit
Tear size	6.2	0.359	25.916	1	0.000^*^	−2.528	−1.123
Fatty degeneration							
0	0.11	1.457	2.326	1	0.127	−0.634	5.078
1	0.27	1.364	0.926	1	0.336	−1.361	3.985
2	0.36	1.392	0.532	1	0.466	−1.712	3.743
3	0.996	1.466	0.000	1	0.998	−2.869	2.876
4	1	–	–	0	–	–	–
Pain							
1	0.21	1.958	0.654	1	0.419	−2.254	5.422
2	0.27	1.461	0.785	1	0.376	−1.568	4.157
3	0.15	1.608	1.354	1	0.245	−1.280	5.023
4	0.75	1.321	0.046	1	0.829	−2.304	2.873
5	2	1.341	0.269	1	0.604	−3.322	1.933
6	4.57	1.382	1.209	1	0.272	−4.229	1.190
7	1.67	1.404	0.134	1	0.715	−3.266	2.239
8	3.62	1.491	0.746	1	0.388	−4.209	1.634
9	2.92	2.132	0.252	1	0.616	−5.248	3.109
10	1	–	–	0	–	–	–

*P<0.001.

### Evaluation of the Value of Upward Migration Index for Predicting Rotator Cuff Tear by the Receiver Operator Characteristic Curve

**Figure 2:**
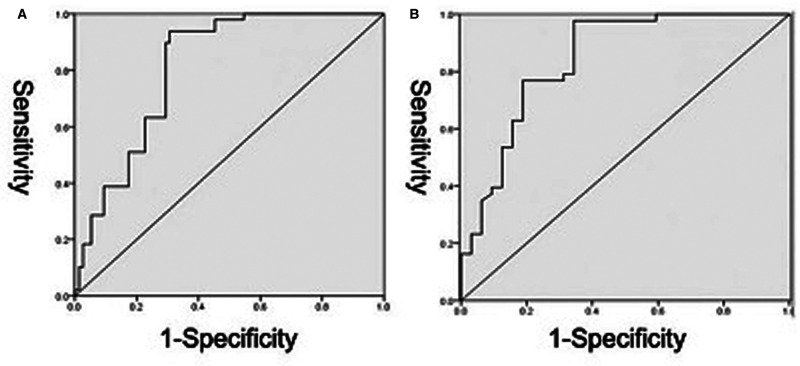
ROC curve to determine the cutoff value of UMI for discriminating tear (**A**) and tear size (**B**). The reference line is indicated in green. The values of area under the curve were 0.798 and 0.843, respectively.

The UMI values of 52 patients without rotator cuff tears, together with the 75 patients with rotator cuff tears, were used as test variables, and the presence or absence of rotator cuff tear as state variables to draw ROC curves (**Figure 2A**). By analyzing the sensitivity and specificity at different cutoff points, the points with the largest Youden index were selected as the diagnostic criteria to determine the rotator cuff tear. Hence, the cutoff value was 1.38, as described in **Table 6**.

**Table 6:** Results of ROC analysis on tear and UMI.

3 Factors 6 UMI	AUC	95% CI	Youden’s index	Cutoff value	*p*-value
UMI (a)	0.798	0.722–0.874	0.597	1.38	<0.01
UMI (b)	0.843	0.749–0.938	0.633	1.30	<0.01

When the UMI of the 75 patients with rotator cuff tears worked as the test variable and the tear size classified as 200 mm^2^ as the state variables, the ROC curve was drawn (**Figure 2B**). Similarly, the point with the largest Youden index was selected as the diagnostic criterion to determine the rotator cuff tear size; the cutoff value was 1.3 (**Table 6**).

## Discussion

Superior rotator cuff tears can lead to superior humeral migration and subacromial impingement (14). The dynamic changes of the glenohumeral joint occur in the progressive stage of a rotator cuff tear and indicate a poor prognosis (15). However, the natural process and related factors involved in these changes have not been clarified yet. The current judgment of proximal humeral migration mainly depends on the direct measurement of AHD. An arthroscopic study found that the average distance between the humeral head and acromion was 10.5 mm. When the rotator cuff torn, the average distance was reduced to 8.2 mm (16). AHD < 7 mm predicted a larger rotator cuff tear and a dramatic decrease in the success rate of surgical repair (4, 17). Therefore, 7 mm is a widely accepted criterion. However, because of the anatomical differences and X-ray magnification on subacromial spaces, the measurement of AHD directly by an X-ray examination is thought inaccurate. UMI was introduced to evaluate humeral upward migration more reliably and accurately by eliminating the influence of positional and anatomical differences and other factors. By the single-factor correlation analysis, many variables, especially CA and *R*, were related to age, sex, and BMI, while UMI had no correlation with gender, age, BMI, etc. Therefore, the novel index, UMI = CA/*R*, was introduced in this study to effectively eliminate the confounding impact of the above factors, which provided a new judging reference for the proximal humeral migration in the future.

The main purpose of this study is to explore the factors resulting in the upward migration of the proximal humerus and analyze their contributions. First, Pearson correlation analyses were carried out, respectively, between the influencing factors and UMI, demonstrating that the tear size of the supraspinatus/infraspinatus muscle, fatty degeneration grade, and VAS score were correlated with the UMI grade. Namely, the larger the tears or more severe the fatty degeneration, the greater the migration of the proximal humerus. To further verify the conclusions, the VAS score, the logarithm of tear size, and the fatty degeneration grade were selected as independent variables and the UMI grade as the dependent variable for ordinal multinomial logistic regression analysis. It suggested that the tear size was an independent factor among all factors; namely, a larger tear size indicated a lower grade of UMI and more severe migration of the proximal humerus. Additionally, when the UMI value was <1.3, more than 90% of patients were found to have a large full-thickness tear (over 200 mm^2^). However, there were exceptions in this study: a few patients with UMI values >1.3 also displayed large tears, indicating that although the reduced UMI value could reflect the integrity of supraspinatus/infraspinatus, it should not be the only criterion for judging tear severity and treatment options.

In the past decades, many studies emphasized the significant role of early diagnosis and treatment in the improvement of the cuff repair effect (18, 19). Ultrasound and MRI provide a highly specific and accurate diagnosis of the rotator cuff tear, which makes them the main imaging examination. However, in clinical practice, an X-ray is always the first examination in the case of acute shoulder pain because of its convenience and intuitive feature of the bony structure. Hence, the preliminary judgment from the early X-ray is critical for the diagnosis and further treatment. Our study indicated a significant correlation between the UMI and tear size, which partially verified the MRI examination results. Therefore, in the primary outpatient and emergency diagnosis, the reduced UMI value in an X-ray with positive physical examination signs can preliminarily determine the degree of rotator cuff tear. Moreover, when the UMI value is <1.3, a wide range of tear should be highly suspected, which works as an important reference for the early and accurate diagnosis. However, many other factors are also involved in the final choice of treatment and prognosis, such as age, muscle strength, gender, and functional needs, which require surgeons to combine multiple factors to make a judgment, such as the medical history and physical and other examinations.

With regard to the factors affecting the upward migration of proximal humerus, Keener et al. studied the effect of tear size and pain on the dynamics of the glenohumeral joint (20). They found that in addition to size, pain was also associated with the upward migration of the proximal humerus. However, Yamaguchi et al. found that symptomatic and asymptomatic patients with rotator cuff tears both displayed an upward migration of the proximal humerus (18). Hence, the effect of pain is still controversial. For the relationship between fatty degeneration and proximal humeral displacement, Nové-Josserand et al. found that the subacromion space was narrower in the fatty degeneration cases (21). However, normal subacromial space was found in some patients with grade 2 or grade 3 fatty degeneration in our study. Although univariate analysis showed that the fatty degeneration grade and VAS score were correlated with the UMI grade, the correlation was weaker than previously reported. Further ordinal multinomial logistic regression analysis confirmed that the two factors could not affect UMI. The specific reason for this difference from previous research is still unclear, which requires further multicenter clinical trials to verify in the future.

Our results suggest that there is no correlation between the tendon thickness at the tear site and the UMI. This may result from the tendon retraction after a rotator cuff tear and the tissue adhesion around the tear site, which result in uneven thickness. Since the tendon itself has a certain elasticity, the measurement value may be uncertain to some extent. Thus, the UMI on radiographs is a good indicator in the clinical diagnosis of supraspinatus/infraspinatus injury. According to the ROC curve, UMI < 1.38 implicated a normal tear, while a large tear was highly suspected when UMI < 1.3.

There are some limitations to this study. First, this study mainly focused on full-thickness tears, and the changes of partial tears were not included, which should be further studied in the future. Eventually, the conclusion in this study is still theoretical and needs further biomechanical experiments and clinical verification in the future.

## Data Availability

The raw data supporting the conclusions of this article will be made available by the authors, without undue reservation.
